# Three-Dimensional Numerical Simulation of Grain Growth during Selective Laser Melting of 316L Stainless Steel

**DOI:** 10.3390/ma15196800

**Published:** 2022-09-30

**Authors:** Feng Xu, Feiyu Xiong, Ming-Jian Li, Yanping Lian

**Affiliations:** Institute of Advanced Structure Technology, Beijing Institute of Technology, Beijing 100081, China

**Keywords:** additive manufacturing, selective laser melting, grain structure, keyhole mode melting, multi-physical simulation

## Abstract

The grain structure of the selective laser melting additive manufactured parts has been shown to be heterogeneous and spatially non-uniform compared to the traditional manufacturing process. However, the complex formation mechanism of these unique grain structures is hard to reveal using the experimental method alone. In this study, we presented a high-fidelity 3D numerical model to address the grain growth mechanisms during the selective laser melting of 316 stainless steel, including two heating modes, i.e., conduction mode and keyhole mode melting. In the numerical model, the powder-scale thermo-fluid dynamics are simulated using the finite volume method with the volume of fluid method. At the same time, the grain structure evolution is sequentially predicted by the cellular automaton method with the predicted temperature field and the as-melted powder bed configuration as input. The simulation results agree well with the experimental data available in the literature. The influence of the process parameters and the keyhole and keyhole-induced void on grain structure formation are addressed in detail. The findings of this study are helpful to the optimization of process parameters for tailoring the microstructure of fabricated parts with expected mechanical properties.

## 1. Introduction

Metal additive manufacturing (AM) presents significant advantages in fabricating complex parts, freeing the designers from traditional manufacturing constraints and offering a way to tailor the microstructure and thereby build physical and mechanical properties. It has been attracting tremendous attention in many fields [1,2,3], such as aerospace, transportation, and biomedical engineering. Many metal-AM techniques exist, such as powder bed fusion and directed energy deposition processes. Among them, selective laser melting (SLM) is one of the most widely used powder bed fusion AM processes for manufacturing fine-detailed parts with high dimensional accuracy [4]. However, the SLM still suffers from many issues relating to the formation of complex grain structures and manufacturing defects in fabricating parts with expected properties [5]. To overcome these issues, one needs to have an in-depth understanding of the relationship between the process parameters and the microstructure evolution during solidification from the single-track to multi-layer depositions with various scan strategies and the interplay between the defect (i.e., porosity) and the complex grain structure [6,7].

This study focuses on the solidification microstructure of 316L stainless steel printed using the SLM. Much effort has been devoted to the experimental study of the grain structure of the printed steel alloys under different process parameters. Liverani et al. [8] conducted the experiments to investigate the correlation between the process parameters (e.g., laser power, build direction, hatch spacing) and the resulting microstructure and mechanical properties of SLM 316L specimens. It is identified that the sample’s mechanical behavior is comparable or superior to that of the reference material, thanks to its unique microstructure. Kurzynowski et al. [9] fabricated high-density 316L stainless steel samples by SLM with different process parameters. It is shown that laser energy density and scan strategy strongly influence the resulting microstructure, and the specific grain structure in as-built conditions can increase the yield strength of the 316L specimen. In addition, Montero-Sistiaga et al. [10] focused on the laser power effect on the morphological and crystallographical texture and grain size. It is found that high laser power (e.g., 1 kW) can generate columnar grains along the building direction, while low laser power (e.g., 400 W) can yield a finer sub-grain microstructure. Recently, Bang et al. [11] further addressed the influence of laser energy density on microstructure and mechanical properties of 316L stainless steel parts by SLM over 36 specimens. It is again confirmed that the grain size is increased in proportion to the energy density. The effect of scan strategy on the microstructure, grain growth, grain size, and mechanical properties of SLM-formed 316L stainless steel were also addressed in [12,13]. Although many works, as mentioned above, show the influence trend of process parameters on microstructure only via the cross-section view of the resulted grain structure, the correlation between the process parameters and microstructure is still not well understood. This is because, compared with that of the traditional manufacturing process, e.g., casting and welding, the microstructure of SLM additive manufactured builds presents characteristics of heterogeneous and spatial variation associated with the complex raster pattern with high cooling rate and temperature gradient [14,15]. Moreover, it is time consuming and expensive to conduct the experimental study of the resulted 3D grain structure. Therefore, using 3D numerical methods to address the underlying mechanisms of the grain growth is significantly meaningful and important from single-track-by-single-layer to multi-track and multi-layer with different combinations of process parameters.

For the time being, there are many numerical studies of grain growth during metal additive manufacturing. In these numerical studies, the widely used numerical methods are phase field (PF) method [16,17,18,19], cellular automaton (CA) method [20,21,22,23], and Monte Carlo (MC) method [24,25,26,27]. Compared with PF and MC methods, the CA method best balances the computational efficiency and the resolved physical details of grain. To predict the grain structure in AM, CA must be coupled with other numerical methods, such as the finite volume method (FVM) and finite element method, for the heat transfer analysis. Lian et al. [28] proposed a three-dimensional cellular automaton finite volume method for predicting the grain structure of IN718 alloy by direct energy deposition, where the FVM is used for the thermo-fluid flow simulation. The effects of laser scan speed and power on the grain size and morphology of the single-track scanning fusion layer were explored, and the grain growth during a single-track-by-multi-layer deposition under different scanning strategies in the experiment was reproduced. Xiong et al. [29] further improved this framework by coupling the discrete element method for powder layer deposition. The β phase microstructure evolution of Ti6Al4V alloy during the multi-track and multi-layer printing of selective electron beam melting process was predicted, and the formation process of complex microstructure in overlap zone was revealed for the first time thanks to the proposed high-fidelity model. For 316L alloy, Zhang et al. [30] proposed an integrated framework combining computational fluid dynamics and cellular automaton and addressed the influence of laser scan speed on the grain structure and orientation during the SLM process. Zinovieva et al. [31] applied the CA for grain structure prediction of 316L stainless steel printed by SLM, where the temperature field of the melt pool is assumed to stay unchanged and then solved during preprocessing for a single track. The numerical results demonstrate that the unidirectional scan pattern tends to yield coarser grains with stronger texture than those printed using a bidirectional scan pattern. In these studies focusing on the CA predictions of 316L stainless steel, most considered the powder bed as a continuity material rather than one that resolved the powder particles. However, the predicted melt pool should be of realistic size and shape, and the temperature field within the melt pool and mushy zone should be as realistic as possible to predict microstructure evolution during the SLM process. Therefore, the numerical simulation via the coupling of CA and the powder-scale thermo-fluid flow model is required to understand complex microstructure evolution during solidification from the single-track to multi-layer depositions with various raster patterns. There are two types of melting modes during laser melting. They are called conduction mode (with melt pool aspect ratio below 0.8) and keyhole mode melting. In the SLM process, the keyhole often appears under the condition of high laser power and low scanning speed [7,32]. However, most of the work was devoted to the conduction mode melting, where the melt pool with an aspect ratio of below 0.8, and little work was devoted to the keyhole model melting with a melt pool aspect ratio (width to depth) above 0.8. Moreover, there is little numerical work on the effect of keyhole-induced porosity in grain structure development of 316L stainless steel during the keyhole mode melting process.

This study aims to numerically address the process-parameters-dependent grain structure of 316L stainless steel fabricated by SLM using a 3D high-fidelity numerical model. We applied the discrete element method (DEM) to build the powder layer model with randomly distributed metal particles. With the DEM result as input, we used the finite volume method and volume of fluid (VOF) method to solve the thermo-fluid flow problems from single-track to multi-track-by-multi-layer deposition. Finally, we applied CA to predict the grain structure with the powder-scale simulation results of the melt pool and thermal field as input. Experimental results from the literature demonstrate the accuracy and effectiveness of the proposed model. After the validation, the effects of the laser power, scan speed, and scan strategy on the grain structure are presented under the conduction mode melting. For the keyhole mode melting, the keyhole with voids is presented, and their effect on the grain structure formation is addressed in depth.

## 2. Methodology

The selective laser melting process includes complex physical phenomena such as heat transfer, mass transfer, and phase transformation associated with the powder bed. In order to accurately simulate the melt pool dynamics and solidification process, we used an integrated modeling framework proposed in our previous work [29]. This framework consists of three models. A powder spreading model is first used for each powder layer to determine the spread powder geometry. Next, a powder-scale thermo-fluid flow model simulates the powder bed melting. After obtaining the transient temperature field and as-melted powder bed configuration, we adopted a CA model to predict the as-built grain structure of 316L stainless steel. The details of these models are described in our previous paper [29], and the salient features of the thermo-fluid flow model and CA model are presented in the following subsections.

### 2.1. Thermo-Fluid Flow Model

**Figure 1:**
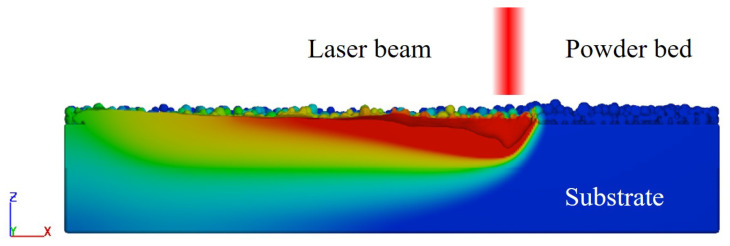
Thermo-fluid flow model.

The three-dimensional temperature field and powder bed geometry evolution during SLM of 316L stainless steel are solved by the 3D heat transfer and fluid flow model using the FVM with the VOF method in the Eulerian description. As shown in Figure 1, the global coordinate system is *X*-axis positive in the laser scanning direction, *Z*-axis positive in the build direction, and *Y*-axis positive in the direction perpendicular to the XZ plane. The numerical model solves the continuity equations, momentum conservation equations, and energy conservation equations to obtain the temperature and velocity fields, melt pool, and multiple thermal cycles during the multi-track, multi-layer deposition process. The governing equations are described as follows.

Continuity equation:

(1) $$\frac{\partial \rho }{\partial t}+\nabla \cdot \left(\rho u\right)=0$$

where *t* is the time, ρ is the density, and u is the velocity.

Momentum conservation equation:

(2) $$\frac{\partial }{\partial t}\left(\rho u\right)+u\cdot \nabla \left(\rho u\right)=\nabla \cdot \left(\mu \nabla u\right)-\nabla p-\rho g\beta \left(T-T_{r}\right)-K_{0}\frac{{\left(1-f_{l}\right)}^{2}}{f_{l}^{3}}u+\rho g$$

where μ is dynamic viscosity, and *p* represents the pressure. The third term on the right-hand side (RHS) of Equation (2) is the buoyancy term based on Boussinesq approximation, β is the thermal expansion coefficient, $T_{r}$ is the liquidus temperature, and g is the gravity acceleration. The fourth term on the RHS of Equation (2) is the Darcy term, which represents the damping force of fluid in the mushy zone, $f_{l}$ represents the volume fraction of fluid, $K_{0}$ represents the permeability of fluid flow and is determined by the Kozeny–Carman formula [33].

Energy conservation equation:

(3) $$\frac{\partial }{\partial t}\left(\rho H\right)+u\cdot \nabla \left(\rho H\right)=\nabla \cdot \left(k\nabla T\right)-\frac{\partial }{\partial t}\left(\rho \Delta H\right)-u\cdot \nabla \left(\rho \Delta H\right)$$

where *H* is the enthalpy of material, *k* is heat conductivity, and ΔH is the enthalpy of solid–liquid phase change.

To close the above-mentioned conservation equations, one requires the boundary conditions and initial conditions. For the boundary conditions at the metal/gas interface, recoil pressure and surface tension are applied as follows.

(4) $$f_{r}=0.54p_{0}\exp\left(\frac{L_{v}m}{k_{B}}\left(\frac{1}{T_{v}}-\frac{1}{T}\right)\right)n$$

(5) $$f_{s}=\sigma n\kappa +\frac{d\sigma }{dT}\left[\nabla T-n\left(n\cdot \nabla T\right)\right]$$

where $f_{r}$ represents the recoil pressure on the surface of the melt pool corresponding to the vaporization of metal materials, $p_{0}$ is environmental pressure, $L_{v}$ is the latent heat of vaporization, *m* is the molecular mass, $k_{B}$ is the Boltzmann constant, $T_{v}$ is the boiling temperature, and n is the unit normal vector. $f_{s}$ represents the surface tension, σ is the surface tension coefficient, $\frac{d\sigma }{dT}$ is the temperature coefficient of surface tension, and κ is the curvature.

At the interface of metal and gas, the boundary conditions of the energy conservation equation are given as follows:

(6) $$-k\frac{\partial T}{\partial n}=h_{c}\left(T-T_{ref}\right)+\sigma_{s}\varepsilon \left(T^{4}-T_{ref}^{4}\right)+h_{v}+q$$

where $h_{c}$ is the convective heat transfer coefficient, $T_{ref}$ is the reference temperature, $\sigma_{s}$ is the Stefan–Boltzmann constant, and ε is the emissivity. The heat flux of evaporation energy loss [34], $h_{v}$, is calculated as:

(7) $$h_{v}=0.82\frac{L_{v}m}{\sqrt{2\pi mk_{B}T}}p_{0}\exp\left[\frac{L_{v}m}{k_{B}}\left(\frac{1}{T_{v}}-\frac{1}{T}\right)\right]$$

Due to the high energy input of selective laser melting, the liquid metal over the melt pool surface evaporates, and the keyhole will appear under the action of recoil pressure. At this time, the laser will reflect multiple times on the surface of the keyhole. Conventional surface heat source models often underestimate laser energy input, so a ray tracing model is needed to simulate the laser heat source. The laser beam is first decomposed into $N_{ray}$ rays, which are determined by the spatial resolution of the grid. The total laser energy input *q* is then calculated by:

(8) $$q=\sum_{i=1}^{N_{ray}}\sum_{j=1}^{N_{inc}}q_{i,j}\eta_{i,j}$$

where $q_{i,j}$ is the energy of the *i*th ray before its *j*th incident. $N_{inc}$ is the maximum allowed number of reflections for each ray. The absorption rate is evaluated by $\eta_{i,j}=\eta_{0}\cos\theta_{i,j}$ with $\eta_{0}$ being the maximum absorption rate and $\theta_{i,j}$ the corresponding incidence angle. The relationship between reflection direction R, incident direction I, and normal direction N is expressed as R=I+2(−I·N)N. In this work, assuming that the laser heat flux follows a two-dimensional Gaussian distribution, the initial energy $q_{i,1}$ of each ray is given by the following formula:

(9) $$q_{i,1}=\frac{2P}{\pi r^{2}}\exp\left[-2\frac{{\left(x-x_{0}-v_{l}t\right)}^{2}+{\left(y-y_{0}\right)}^{2}}{r^{2}}\right]$$

where *P* is the laser power, *r* is the radius of laser spot, $\left(x_{0},y_{0}\right)$ is the initial horizontal coordinate of the spot center, $v_{l}$ is the laser scan speed given along the *X*-axis, and $q_{i,j+1}$ is calculated as:

(10) $$q_{i,j+1}=q_{i,j}\left(1-\eta_{i,j}\right)$$

For other sides of the material domain, the boundary conditions are set as continuative to achieve a smooth continuation of the flow and heat flux through the boundary. The initial conditions are set based on the real process conditions.

The free surface of the melt pool is captured using the VOF method. The volume fraction *F* is transported by the following equation.

(11) $$\frac{\partial F}{\partial t}+\nabla \cdot \left(Fu\right)=0$$

The FVM is employed to solve the momentum and continuity equations using an operator-splitting scheme [35]. The advection term in Equation (2) is discretized by a second-order upwind method [36], and the temporal term is discretized by a first-order Euler method. The pressure is solved by the generalized minimum residual method (GMRES) [37]. In the VOF method, a donor–acceptor approach [38] is applied to evaluate the volume fluxes. Once the fluid field is obtained at each time level, the energy Equation (3) is then solved by a first-order explicit time integration scheme.

### 2.2. Grain Structure Prediction Model

**Figure 2:**
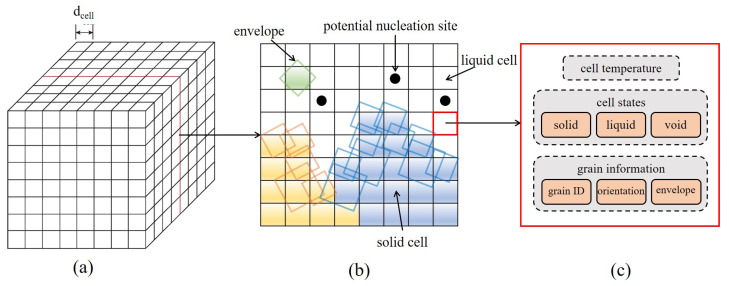
Grain structure evolution model: (**a**) a regular network of cellular automata model, where $d_{cell}$ is the cell size, (**b**) a cross-section view of the network, and (**c**) the information carried by each cell during the simulation.

The three-dimensional 316L stainless steel microstructure evolution associated with the SLM process thermal field is solved by the CA model. In the CA model, the material region is discretized into a set of cubic cells, as shown in Figure 2. Each cell is assigned a set of variables. They are temperature, state label (for solid, liquid, and void corresponding to gas), and grain information (including grain ID, crystallographic orientation, and envelope, introduced later on). The temperature is updated in the simulation using the thermo-fluid flow model and other cell variables associated with the grain nucleation and growth by two sub-models, which are explained in the following subsections.

#### 2.2.1. Nucleation Model

In CA, the location of nucleation sites, activation criteria, and crystal orientation of newly nucleated grains are handled by a nucleation model. In the selective laser melting process of 316L stainless steel, both epitaxial grain growth from the partially melted grains at the melt pool boundary and nucleation within the melt pool are frequently experimentally observed. In order to capture these nucleation phenomena, the enriched nucleation model proposed in our previous paper [28] is used. It consists of bulk nucleation and activation of existing grains along the melt pool boundary.

For bulk nucleation, the total number of potential nucleation sites is determined by an input parameter, nucleation number density $\rho_{v}$. It is usually obtained via fitting experimental measurements. For a given volume of a region, we calculate the total number of nucleation sites $N_{v}$ as follows:

(12) $$N_{v}=\rho_{v}V$$

where *V* is the total volume. In the given discretization domain, $N_{v}$ cells are randomly selected and defined as potential bulk nucleation sites. During the solidification process, for each pre-chosen cell *i*, a new grain is generated if the undercooling at the cell center exceeds the critical value $\Delta T_{i}^{crit}$, which is assumed to follow the Gaussian distribution characterized by mean value $T_{N}$ and standard deviation $\Delta T_{\sigma }$. Then, the state label of the cell *i* is changed from liquid state to solid state, and a unique grain ID is assigned to the cell. The crystallographic orientation associated with the new grain using a set of randomly generated Euler angles $\left(\varphi_{1},\varphi_{2},\varphi_{3}\right)$, where $0\leq \varphi_{1}\leq 2\pi$, $0\leq \varphi_{2}\leq \pi$, and $0\leq \varphi_{3}\leq 2\pi$ is allocated to the cell *i*. Meanwhile, an envelope representing the shape and size of the grain is generated at the center of the cell. More details of the envelope are explained in the following subsection.

For the epitaxial grain growth from the melt pool boundary, the partially melted grains from the substrate or the previous deposition layer act as seeds and continue to grow. During the scan process, if the moving melt pool engulfs the cell with a solid state, its grain information is erased, and its state label is reset to the liquid state. For the unmelted cells at the melt pool boundary under cooling, if they have at least one adjacent neighboring cell in the liquid state and their temperature is higher than the solidus temperature, they are reactivated and keep their inherent grain information as they grow.

#### 2.2.2. Grain Growth Model

**Figure 3:**
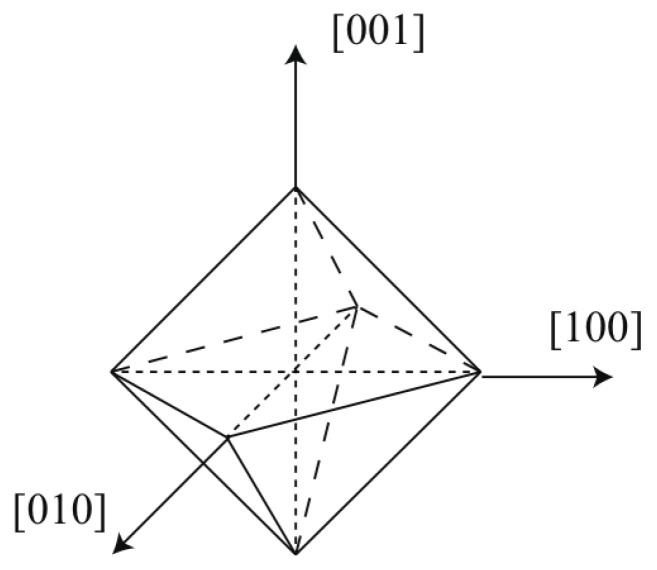
Regular octahedral envelope.

In the CA grain growth model, the grain shape and size are resolved by a combination of envelopes, where the dendritic structure details are ignored. Considering that 316L stainless steel has a face-centered cubic crystal structure, the corresponding envelope is set to have an octahedral shape, as shown in Figure 3. The six half-diagonals represent the preferred <100> crystallographic directions defined by the Euler angles, along which the grain grows fastest [22].

During the solidification process, the grain growth is captured by expending the six half-diagonal envelopes based on the dendrite tip’s growth kinetics. The dendrite tip velocity *v* is related to the local undercooling based on the Lipton–Glicksman–Kurz dendrite tip supercooling model [39,40]. The relationship between the dendrite tip growth velocity *v* and the undercooling ΔT is fitted by polynomial approximation, as follows:

(13) $$v\left(\Delta T\right)=a_{2}\Delta T^{2}+a_{3}\Delta T^{3}$$

where $a_{2}$, $a_{3}$ are the parameters obtained by fitting the experimental data.

For either a new grain or a reactivated grain at cell *i*, a regular octahedral envelope is placed in the center of the cell. Ignoring the incubation time, the half diagonal length of the octahedron envelope at time *t* is calculated by the following formula:

(14) $$L_{i}\left(t\right)=\int_{t_{0}}^{t}v\left[\Delta T_{i}\left(\tau \right)\right]d\tau$$

where $t_{0}$ is the time when the nucleus is activated.

As time proceeds, the octahedron envelope grows up to engulf the center of its neighboring cell. For each captured cell, a new regular octahedral envelope inheriting the grain orientation from the parent envelope is assigned to the cell. However, the center and size of the new envelope are determined via a decentered octahedron method [22]. In this paper, Moore neighbor cell type is adopted, i.e., each inner cell has 26 neighboring cells. If all the neighboring cells are captured, the current cell’s envelope stops growing and is deactivated.

#### 2.2.3. Thermal Field Input

**Figure 4:**
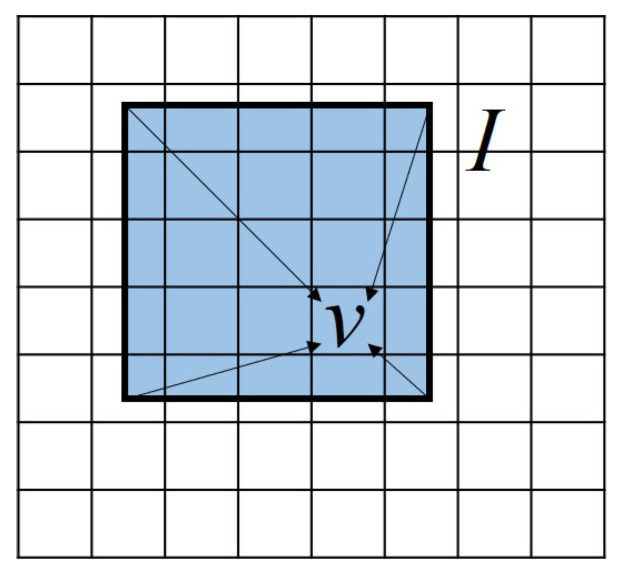
Schematic illustration of the one-way coupling method, taking a 2D case as an example, where the temperature of the finer CA cell ν is interpolated from the coarser FVM cell.

A one-way coupling method is adopted to obtain the thermal field for the CA model from the thermo-fluid flow model. Compared with the thermo-fluid flow model, the CA model requires finer mesh resolution and smaller time steps to resolve the microstructure evolution. Therefore, two sets of mesh with different cell sizes are used for the FVM and CA, respectively. To obtain the temperature field and the material region, we superpose the CA mesh onto the FVM mesh as shown in Figure 4. The CA cell center temperature $T_{\nu }^{t}$ at the time spot of *t* is interpolated from the coarser FVM cell that covers its center, as follows.

(15) $$T_{\nu }^{t}=N_{I}\left(\nu_{c}\right)T_{I}^{t}$$

where ν is the CA cell, *I* is the node of the FVM cell covering the center of the CA cell ν, $\nu_{c}$ is the position of the cell center, $N_{I}\left(\nu \right)$ is the first-order shape function, and $T_{I}^{t}$ is the nodal temperature of the FVM cell. The repeated subscript *I* follows the Einstein summation convention. Using the liner interpolation scheme, we calculate $T_{I}^{t}$ as:

(16) $$T_{I}^{t}=\frac{T_{I}^{n}-T_{I}^{n-1}}{t^{n}-t^{n-1}}\left(t-t^{n-1}\right)+T_{I}^{n-1}$$

where *n* represents the *n*th time step in the FVM simulation, $t^{n-1}$ and $t^{n}$ represent two adjacent time spots, and $t^{n-1}\leq t<t^{n}$ exists. Note that for those FVM cells located in the gap between powder particles or above the powder layer and the melt pool, where no material is present, the corresponding CA cells are labeled as the void state.

## 3. Results and Discussion

Two melting modes of 316L stainless steel fabricated by SLM were conducted to show the process parameter’s effect on the complex grain structure formation. The first set is for the conduction mode melting with different combinations of process parameters. In the conduction mode melting, we first presented the single-track simulations for validation of the integrated modeling framework and the effects of laser power and scan speed. Then, we conducted the multi-track, multi-layer simulations to reveal the development of the complex grain structure in successive tracks and layers with different scan strategies. The second set is for keyhole mode melting with a specific combination of process parameters, where the keyhole with keyhole-induced voids is present. The effect of keyhole mode melting on the microstructure is addressed.

In all the simulations, epitaxial grain growth and bulk nucleation are considered. The initial grain structure for the simulations is assumed to be equiaxed grains with an average equivalent sphere diameter of 3.6 μm. The parameters used in the numerical simulation are shown in Table 1, Table 2 and Table 3. DREAM3D [41] is used to analyze the solidification grain structure.

### 3.1. Experiment Settings and Data

**Figure 5:**
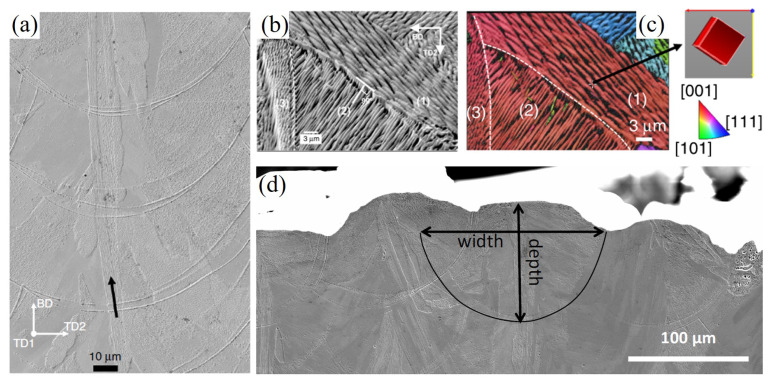
Microstructure development in AM 316L stainless steel A using bi-directional scan without rotation: (**a**) The continuous growth of grains in a slender domain (highlighted by a black row) along the centreline across melt pools, (**b**) cells in the region labeled (3) epitaxially grew from ones in the region (2) which did grow from existing cells in the region (1), (**c**) the corresponding inverse pole figure of grains in (**b**) indicating that the cells in regions labeled (1), (2), and (3) belong to the same grain due to epitaxial growth, but have 90° changes in the growth direction, (**d**) melt pools on the top layer of a 316L build with measured dimensions are 90 ± 20 μm in depth and 145 ± 30 μm in width. The dashed lines in (**b**,**c**) denote the melt pool boundary. (This figure is reproduced from [6]).

The experiment conducted by Pham et al. [6] was taken as a reference for numerical models’ validation. In the experiment, a Renishaw AM250 printer was used to melt the 316L stainless steel powder onto existing solidified layers. The process parameters were optimized to minimize porosity and were as follows: laser power of 180 W, exposure time of 110 μs, point distance of 65 μm, layer thickness of 50 μm, hatch spacing of 125 μm, and laser spot size of about 65 μm. An argon atmosphere was used to protect the material from oxidation. The 316L stainless steel powder has a size distribution, as in Table 4. Multi-layer builds were printed to provide samples using a bi-directional scan without rotation, and the as-built microstructure is shown in Figure 5, where the melt pool size and shape are also provided. Please refer to the reference [6] for more information on the SLM experiment.

### 3.2. Conduction Mode Melting Process

In the following subsections, we first present the validation of reference case for both the melt pool and grain structure predictions. Next, we reveal the laser power and scan speed effects on grain structure via single track cases listed in Table 5, and finally, we address the scan strategy effect on grain structure. For all the single track cases, the region size is set to 1.5 mm × 0.5 mm × 0.18 mm for thermo-fluid flow simulation, of which the 0.4 mm × 0.25 mm × 0.16 mm with a well-developed melt pool proceeding is for the CA simulation. For the multi-track multi-layer cases, the region size is set to 1.5 mm × 0.5 mm × 0.26 mm for thermo-fluid flow simulation, of which the 0.4 mm × 0.5 mm × 0.22 mm with a well-developed melt pool proceeding is for the CA simulation.

#### 3.2.1. Validation via the Single-Track Case

**Figure 6:**
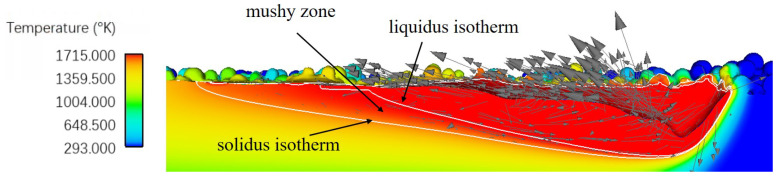
Longitudinal cross-section (XZ plane) view of a single track simulation result, where the white curves represent the solidus and liquidus isotherms, and the arrows represent the flow field within the melt pool.

**Figure 7:**
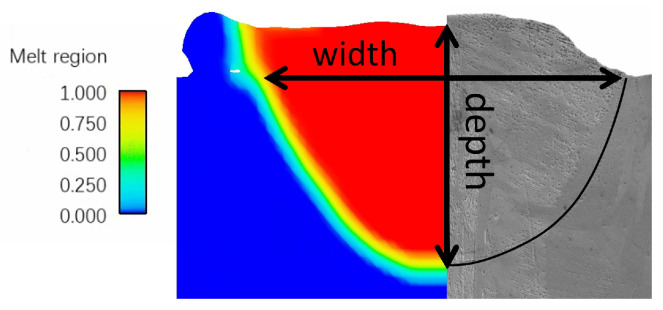
Transverse cross-section (YZ plane) view of the single laser track. The left part is from simulation result and the right part from experiment data.

Following the process parameter settings described in the reference [6], a case ( Case P180V63 in Figure 5) with the laser power of 180 W, the scan speed of 63 cm/s, the layer thickness of 50 μm, and the powder size distribution of Table 4 is first conducted. Figure 6 shows a central longitudinal cross-section view of the thermo-fluid flow simulation result, where the white curves of liquidus and solidus isotherms outline the mushy zone, and the arrays represent the flow field within the melt pool depicted by the solidus isotherm. It is clear that the longitudinal cross-section shape of the melt pool is close to the droplet shape, which is related to the flow pattern driven by the Marangoni effect. From the observation of Figure 6, one can find the backward flow trend on the surface of the melt pool, indicating the outward fluid flow. Meanwhile, there is a depression zone under the laser beam center. It is caused by the recoil pressure and the Marangoni effect. Overall, the predicted temperature and flow fields are in reasonable agreement with the experimental observations [46,47]. To quantitatively validate the thermo-fluid flow simulation result, we plot the transverse cross-section views of the predicted melt pool and the experimental image in Figure 7 and compare the depth and width of the melt pool results in Table 6. The relative error of less than 4% demonstrates that the simulation results and the experimental data agree well.

**Figure 8:**
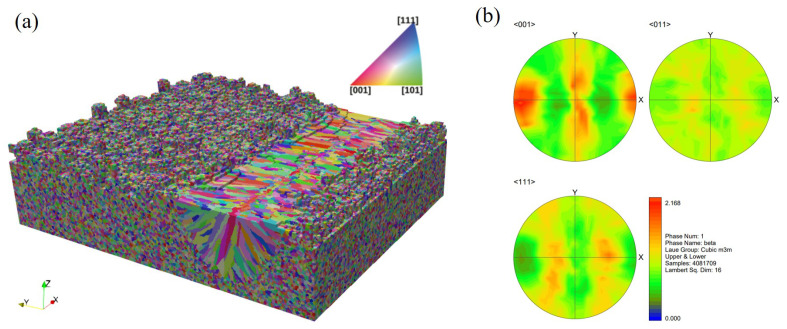
Microstructure simulation results for Case P180V63: (**a**) 3D view, (**b**) pole figures (PF) of the solidification grain structure within the fusion zone.

The 3D view of the final simulated grain structure for Case P180V63 is provided in Figure 8, where the initial grain structure of the unmelted region remains. Grain orientations for simulation results are denoted by an inverse pole figure (IPF) map with a color key, as shown in the insert of Figure 8. The pole figure (PF) of the solidification grain structure within the fusion zone is plotted in Figure 8b. From the observation of Figure 8a, one can find that for the given process parameters, the melt pool is dominated by the columnar grains, while a few grains from the bulk nucleation appear at the top of the melt pool. Such a grain structure morphology is consistent with the profile of morphology factor, which is provided in the following subsection.

**Figure 9:**
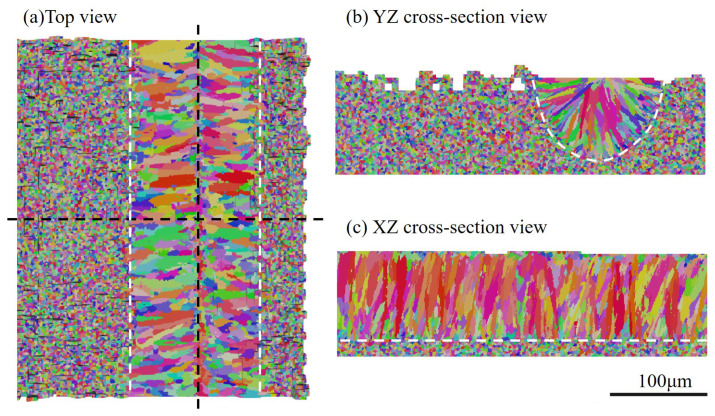
Several 2D views of the single-track simulation results: (**a**) top view of CA result, (**b**) transverse cross-section map of CA, and (**c**) longitudinal cross-section map of CA result, where white dotted lines represent the boundary of the melting zone and black dotted lines represent the locations of the cross-section.

To better probe the spatial distribution of the grains, we plot three 2D views of the simulation results in Figure 9. Figure 9a presents the top view of the grain structure, where the white dotted lines represent the boundary of the fusion zone and black dotted lines denote the locations of the longitudinal and transverse cross-section. The columnar grains from both sides of the melt track proceed in a curved shape towards the center of the melt pool. However, these columnar grains are blocked by the fine grains in the sub-region of the center-line, most of which are demonstrated to be the ones from bulk nucleation (see Figure 9b). The transverse cross-section view of the predicted grain structure is plotted in Figure 9b. It can be observed that the columnar grains epitaxially grow from the partially melted grains located on the fusion line, as indicated by the white dashed lines. Since the growth direction of these columnar grains follows the local temperature gradient direction [28], a radial growth pattern is formed, as shown in Figure 9b. All these grain structure morphological characteristics are consistent with the experimental observations from Figure 1 and Figure 4 in Reference [6].

Figure 9c plots the central longitudinal cross-section view of the simulation result. From the observation, one can find the columnar grains epitaxially grow from the partially melted grains in the substrate, as indicated by the white dotted line. Their growth directions are changed to be slightly inclined to the scan direction, indicating that the local thermal gradient can shape the grains in additive manufacturing via the side-branching mechanism [6]. Moreover, most of the grains have a color close to red, representing the crystallographic orientation [001] closely parallel to the build direction according to the inverse pole figure color code in Figure 8a. This is because the grain with its preferred growth direction best aligned with the temperature gradient is most likely to survive in competitive growth and grows into the larger one. It is confirmed that thermal gradient dictates not only the grain growth direction, but also the grain texture via the grain growth competition.

#### 3.2.2. Laser Power Dependent GRAIN structure

To reveal the effect of laser power on the grain structure formation, we designed four simulation cases with laser power varying from 140 W to 200 W and other fixed process parameters, as listed in Table 5. The selected laser powers are in the reasonable range of experimental conditions for SLM in reference [6,11]. All other simulation settings are similar to that of Case P180V63.

**Figure 10:**
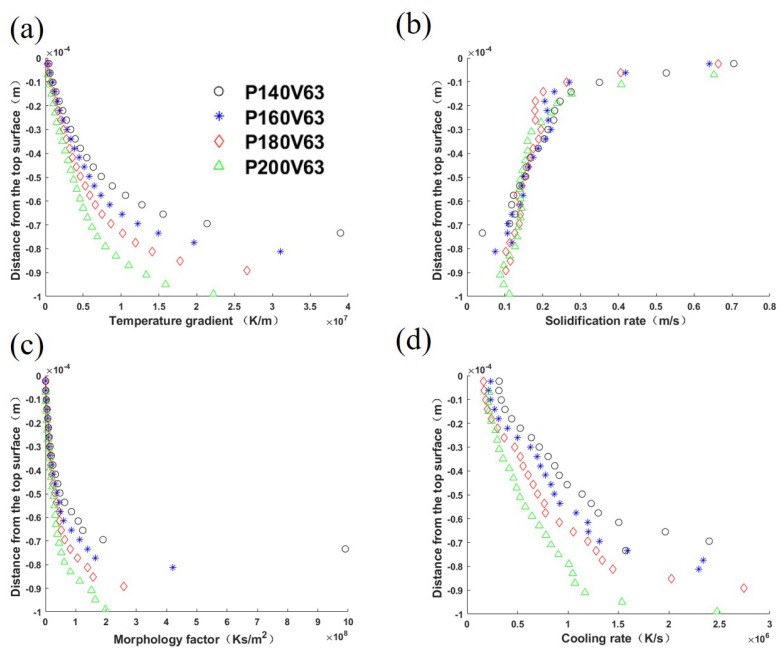
Solidification parameters of the longitudinal section of the melt pool center: (**a**) temperature gradient magnitude *G*, (**b**) solidification rate *R*, (**c**) morphology factor M=G/R, and (**d**) cooling rate C=G·R, for cases with laser power varied within the range [140 W, 200 W], and constant laser scan speed of 0.63 m/s. The abscissa represents the distance from the top surface of the melt pool.

Four thermal features over the mushy zone based on the well-known *G*-*R* map [48] are used to study the solidification mode and grain size. They are temperature gradient *G*, solidification rate *R*, morphology factor *M*, and cooling rate *C*, of which the calculations are detailed in Appendix A. Figure 10 plot theses profiles.

The comparison demonstrates that the temperature gradient magnitude profiles share the same trend, i.e., its value decreases from the bottom of the melt pool to the top surface, as listed in Figure 10a. The laser power variation only brings a slight curve shift among the cases. As shown in Figure 10b, the solidification rate profiles are similar among cases, but increase from the melt pool bottom to the top surface. The morphology factor profiles are plotted in Figure 10c, and follow the temperature gradient profile trend. Consequently, one can expect equiaxed grains from bulk nucleation around the top center of the melt pool. Figure 10d shows that the cooling rate increases from the top surface to the bottom, which corresponds to the mushy zone size variation shown in Figure 6. From the comparisons in Figure 10, it is identified that temperature gradient, morphology factor, and cooling rate decrease with the increase in laser power.

**Figure 11:**
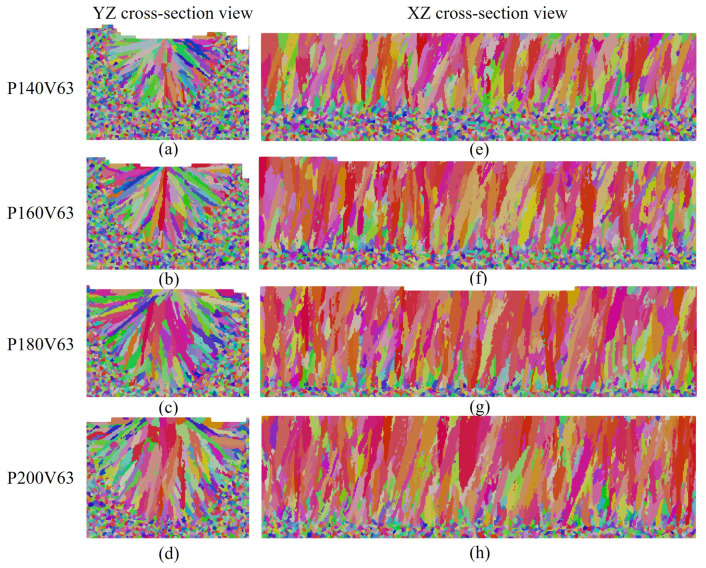
The grain structure cross sections: (**a**–**d**) transverse cross-section map, (**e**–**h**) longitudinal cross-section map of the results for cases with laser power varied within the range of [140 W, 200 W], and the constant laser scan speed of 0.63 m/s.

**Figure 12:**
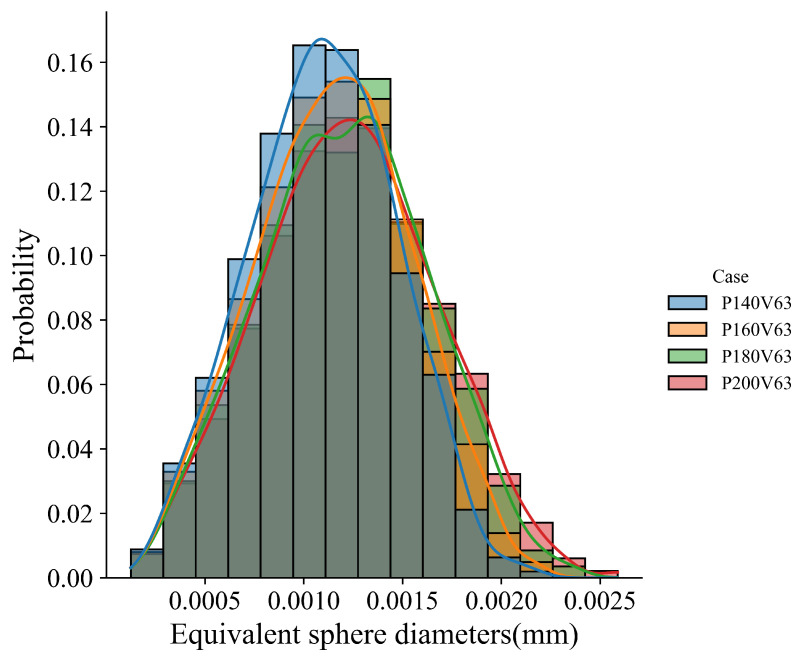
Grain size distributions for cases with laser power varied within the range of [140 W, 200 W] and the constant laser scan speed of 0.63 m/s.

The influence of laser power on grain morphology and size distribution is considered. As shown in Figure 11 for the transverse and longitudinal cross-section views of the four cases, the fusion zone is dominated by the slender columnar grains, and a few equiaxed grains appear in the sub-region of the top center. This demonstrates that the laser power within the given range has little effect on the grain morphology corresponding to Figure 10c. It should be pointed out that columnar grains from epitaxial grain growth dominate the grain morphology of the fusion zone, and therefore the effect of the cooling rate on the grain size within the melt pool for each case is not apparent. However, Figure 12, which compares the grain size distribution curves among the cases, indicates that the grain size becomes finer as the laser power decreases. Such a variation is related to the cooling rate variation among the cases shown in Figure 10d, where the cooling rate decreases with the increase in laser power.

#### 3.2.3. Laser Scan Speed Dependent Grain Structure

To illustrate the relationship between laser scan speed and grain structure development, we designed four simulation cases with laser power varying from 140 W to 200 W and other constant parameters shown in Table 5. The selected laser scan speeds are in the reasonable range of experimental conditions for SLM in reference [6,11].

**Figure 13:**
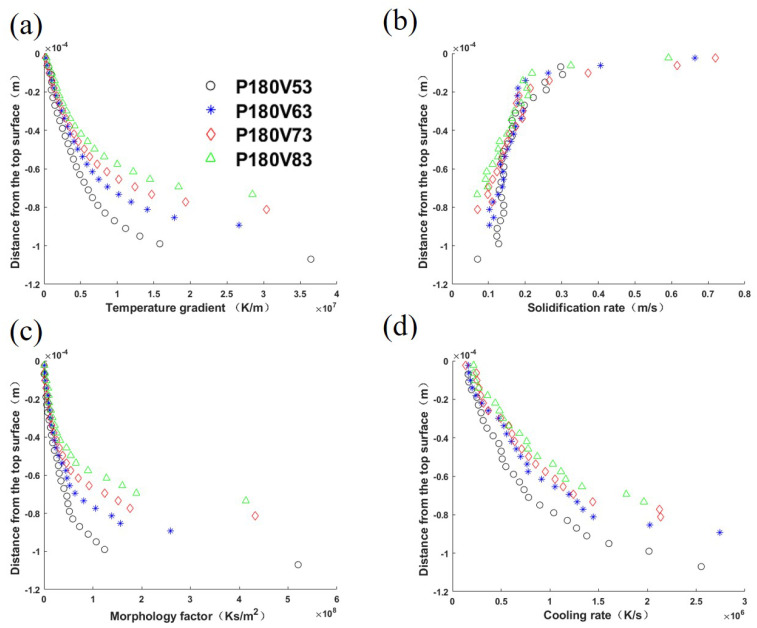
Solidification parameters of the longitudinal section of the melt pool center: (**a**) temperature gradient magnitude *G*, (**b**) solidification rate *R*, (**c**) morphology factor M=G/R, and (**d**) cooling rate C=G·R, for cases with laser scan speed vary within the range of [0.53 m/s, 0.83 m/s], and the constant laser power of 180 W. The abscissa represents the distance from the top surface of the melt pool.

**Figure 14:**
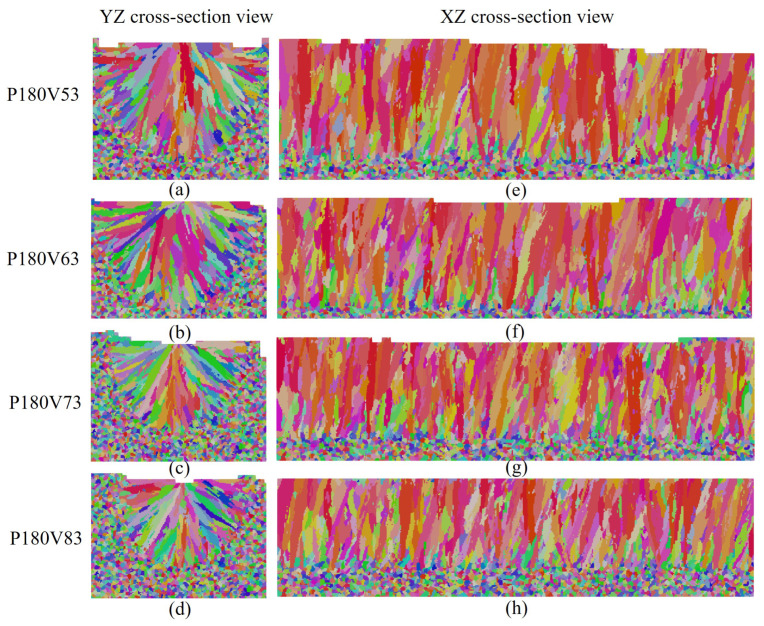
The grain structure cross sections: (**a**–**d**) transverse cross-section map, (**e**–**h**) longitudinal cross-section map of the results for cases with laser power varied within the range [0.53 m/s, 0.83 m/s], and the constant laser power of 180 W.

**Figure 15:**
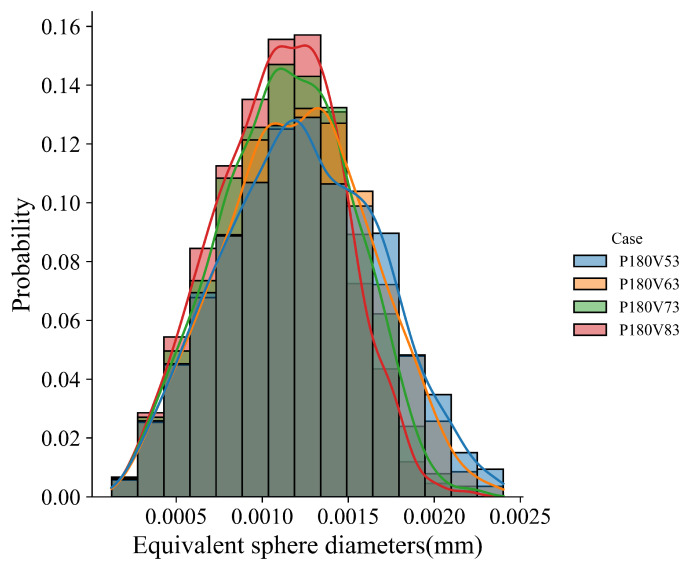
Grain size distributions for the cases with laser scan speed vary within the range of [0.53 m/s, 0.83 m/s] and the constant laser power of 180 W.

The temperature gradient, solidification rate, morphology factor, and cooling rate in the four cases are compared in Figure 13. Figure 13a shows that among the cases, the temperature gradient magnitude increases gradually with the increase in laser scan speed. The solidification rate in most regions increases slightly with the scan speed, but it does not change significantly at the bottom of the melt pool, as plotted in Figure 13b. From the observation of Figure 13c, one can find that the morphology factor almost does not change near the melt pool surface, but increases with the scan speed in the lower part of the melt pool. Figure 13d indicates that the cooling rate increases with the increase in scan speed. Based on these thermal features, one can expect a similar grain morphology among the four cases, as shown in Figure 14, where the transverse and longitudinal cross-section views display the dominant slender columnar grains in the melt pool and a few equiaxed grains near the top center. Figure 15 compares the grain size distribution curves for the four cases. Corresponding to Figure 13d, the grain size distribution shows the trend of grain size decreasing with the increase in scan speed.

#### 3.2.4. Layer-Wise Scan Strategy Dependent Grain Structure

**Figure 16:**
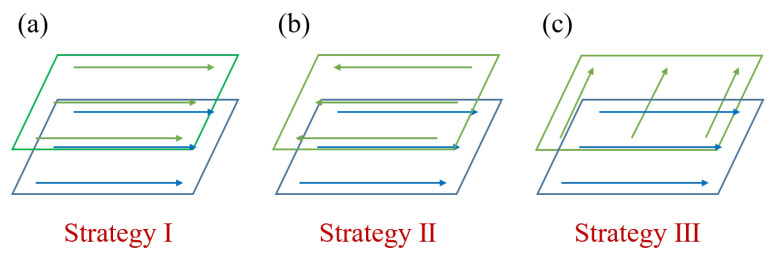
Schematics of layer-wise scan strategy for (**a**) Strategy I: unidirectional scanning without layer-wise rotation, (**b**) Strategy II: unidirectional scanning with layer-wise rotation of 180°, and (**c**) Strategy III: unidirectional scanning with layer-wise rotation of 90°.

In this section, three simulations with different layer-wise scan strategies are conducted to further study the layer-wise scan strategy effect. As shown in Figure 16, they are unidirectional scans without layer-wise rotation, unidirectional scans with 180° layer-wise rotation, and unidirectional scans with 90° layer-wise rotation. For all the cases, the process parameters are the laser power of 180 W, scan speed of 0.63 m/s, hatch spacing of 125 μm, and layer thickness of 50 μm, which are used following the reference [6].

**Figure 17:**
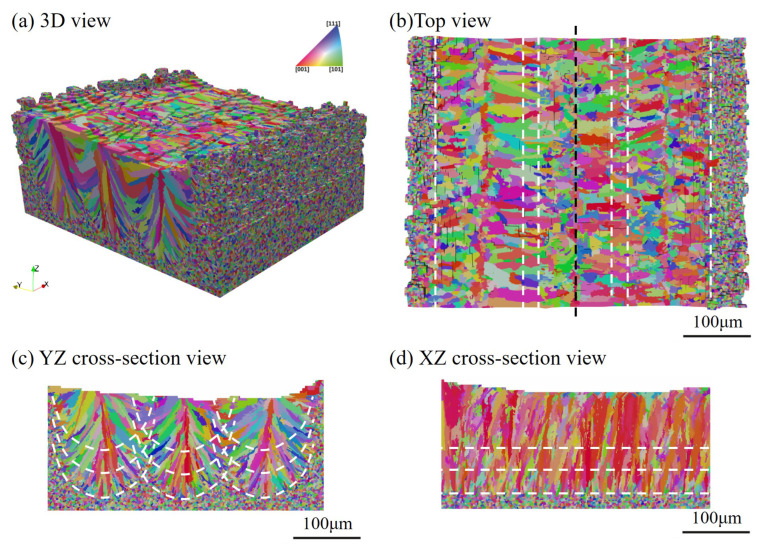
Microstructure simulation results for three-layer and three-track case: (**a**) 3D view of the result, (**b**) top view, (**c**) transverse cross-section map, and (**d**) longitudinal cross-section map, where white dotted lines represent the boundary of the fusion zone, and black dotted lines represent the locations of the cross-section.

**Figure 18:**
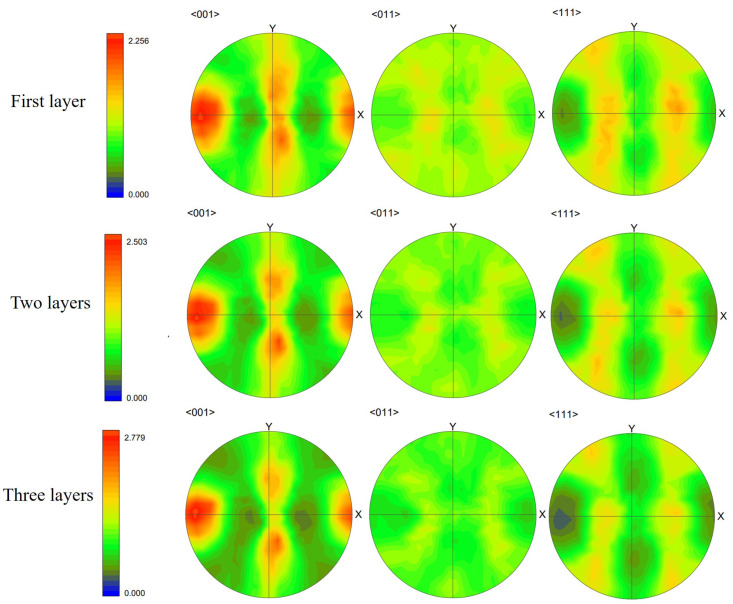
Pole figures (PF) of solidification grain structure within the fusion zone of the simulation results of first-layer, first two layers, and three-layer microstructure.

**Figure 19:**
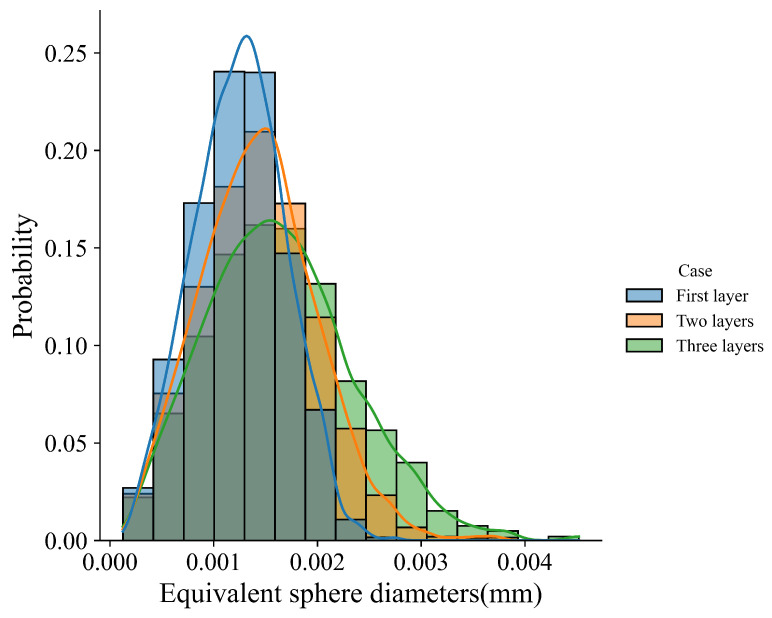
The grain size distribution of the simulation results in the first layer, the first two layers, and the three-layer microstructure.

We first present the simulation results of the case with scan Strategy I. It consists of three layers, and each layer includes three tracks. Figure 17 plots the final grain structure in detail. From Figure 17a for the 3D view of the simulation result, one can find the vertically aligned stacking sequence of melt tracks with more complex grains than those in the single track case. As shown in the top view (Figure 17b) for the third deposited layer, one can find that the inner grains are coarser than those close to the sides, and they straddle the overlap region between neighboring tracks. This is attributed to the competitive grain growth over multi-layer and multi-track under the side-branching mechanism [6], as shown in Figure 17c. From the observation of Figure 17c, one can find that the radial growth pattern exists for each track, as indicated by the white dashed line. However, due to the remelting between the consecutive layer and consecutive track, several unique grain features occur, which are different from those revealed in the single track case. The grains on the sides of the melt pool are re-shaped to be more vertical to the powder bed, while some V-shape coarse grains are present in the track-overlap region outlined by the two intersecting curved white dashed lines in each layer. The grains in the centerline of the tracks keep growing into the subsequent layer deposition without changing growth direction, which is more clearly shown in the longitudinal cross-section view (Figure 17d). This is in good agreement with the result. Moreover, Figure 17d shows most grains in the centerline of the track with a color close to red, indicating strong <001> texture. As demonstrated in Figure 18, the texture of the solidification grain structure gradually becomes stronger with the increase in the deposition layer. The similar temperature gradient pattern among the layers makes the grains preferentially align with the thermal gradient and outgrow unfavorably oriented ones during the melting and remelting of the deposition. Correspondingly, such favorably oriented grains become larger and larger; therefore, the peak of the grain size distributions moves from the upper left to the lower right with the increase in layer depositions, as demonstrated by Figure 19.

To compare the three scan strategies, a basic representative unit of a three-track-by-two-layer is used for twofold concerns. One is that the third layer results in a repeated pattern, similar to how the second layer interacts with the first layer when the remelting depth is less than the depth of the previous fusion zone, as demonstrated in the three-layer simulation result of Strategy I. The other is that the two-layer case is a basic unit used to represent the multi-layer depositions with layer-wise rotation of 180° and 90°.

**Figure 20:**
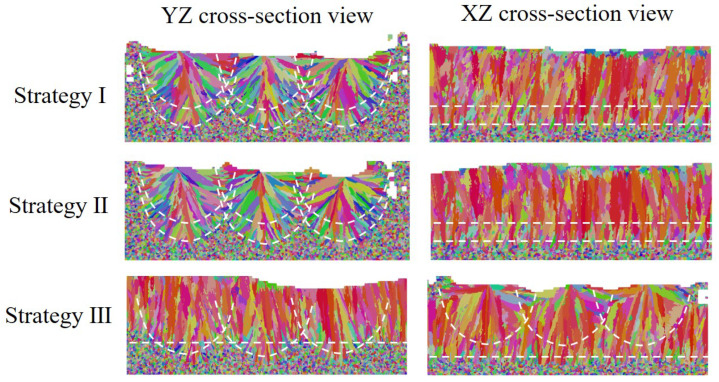
Cross section of simulation results of microstructure evolution under different scanning strategies, where white dotted lines represent the boundary of the fusion zone.

For the three different layer-wise raster patterns, a different alignment of melt tracks and thermal fields along the build direction yields different microstructures, as shown in Figure 20 for 2D views. It is found that the simulation results of Strategy I and II are very similar to each other in the transverse cross-section map, but they are different in the longitudinal cross-section map. In the case of Strategy II, the growth direction of slender columnar grains from the centerline of the track is slightly altered, corresponding to laser scan direction during the second-layer deposition. Moreover, it is identified that the columnar grains confined to the centerline of the melt pool keep growing across melt pools and are nearly vertical, which is in good agreement with experimental data shown in Figure 5a. Because rotating the scan pattern between layers alters the alignment of melt tracks and disrupts the thermal profile along the build direction, the simulation result of Strategy III is significantly different from Strategy I and II. It is found that the columnar grains are still elongated but substantially shortened, which is consistent with the experimental observations in Reference [6].

**Figure 21:**
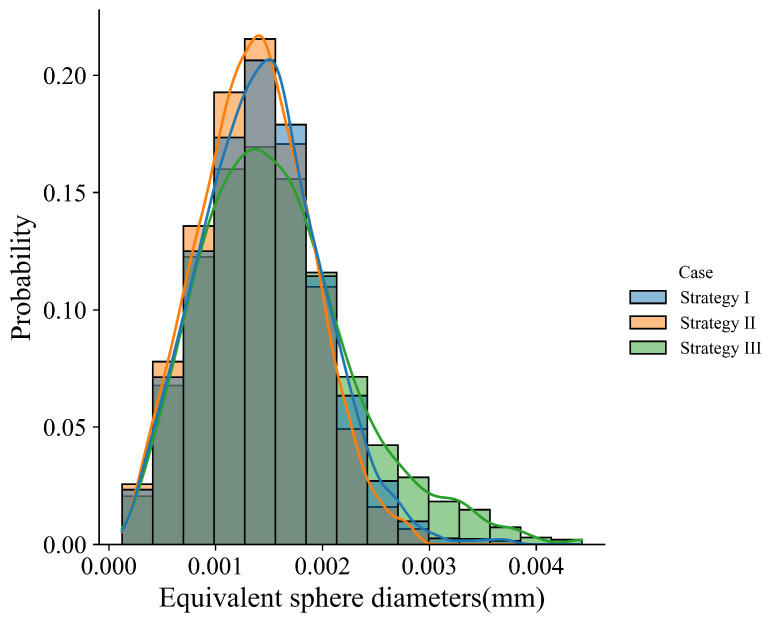
Grain size distribution of the simulation results in the first layer, first two layers, and three-layer microstructure.

**Figure 22:**
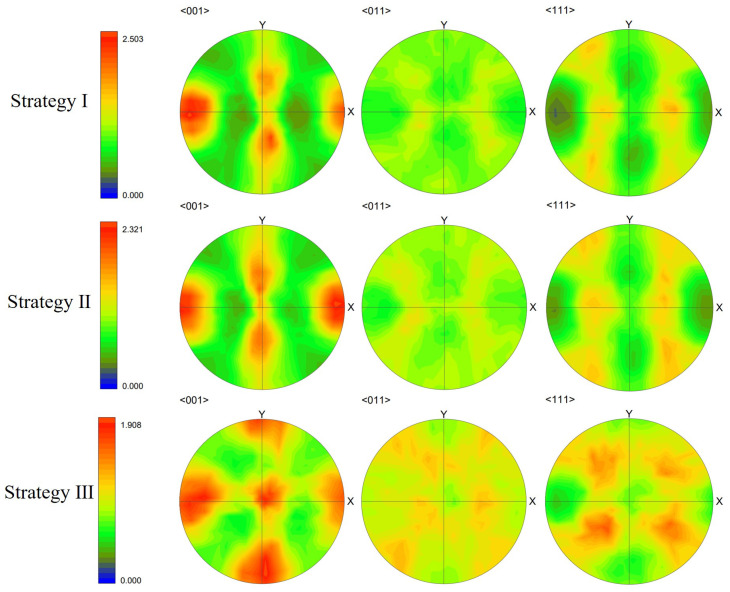
Pole figures (PF) of solidification grain structure within the fusion zone of Strategy I, Strategy II, and Strategy III simulation results.

The layer-wise scan strategy effect on the grain structure formation is further quantitatively measured in terms of grain size distribution and texture intensity. Figure 21 plots the grain size distributions of the three three-track-by-two-layer cases. The comparison shows that the grain size of the Strategy II case is smaller than that of the Strategy I case, i.e., the peak of the former moves to the upper left. However, the difference between Strategy I and Strategy II cases is much smaller than that between Strategy III and the first two strategies. It indicates that the rotation of the interlayer scanning direction by 90° makes more coarse columnar grains present. Figure 22 compares the texture of the three cases for the grain structure within the fusion zone. The obtained texture intensities are 2.503, 2.321, and 1.908 for Strategy I, Strategy II, and Strategy III, respectively. The weak texture trend is related to the disorder degree of the thermal profile, which is dependent on the alignment of melt tracks in the build direction. Moreover, in contrast with the pole figures of the first two strategies, the 90° rotation of scan direction in Strategy III dictates the grains with their <001> orientations aligned with *X*, *Y*, and *Z* directions. In summary, the scanning strategy of rotating the scanning direction between layers can change the arrangement of melting tracks and disrupt the heat distribution along the build direction, which can promote more grains with different orientations to grow.

### 3.3. Keyhole-Mode Melting Process

In this section, we set the laser power (350 W) and the scan speed (0.63 m/s) in a way such that the keyhole mode melting and keyhole-induced pore defect often observed in experiments can be reproduced. For the grain structure prediction, the region size is set to 1500 μm × 400 μm × 1000 μm, and all other numerical settings are the same as in Section 3.2.

#### 3.3.1. Melt Pool with the Presence of Keyhole and Pores

**Figure 23:**
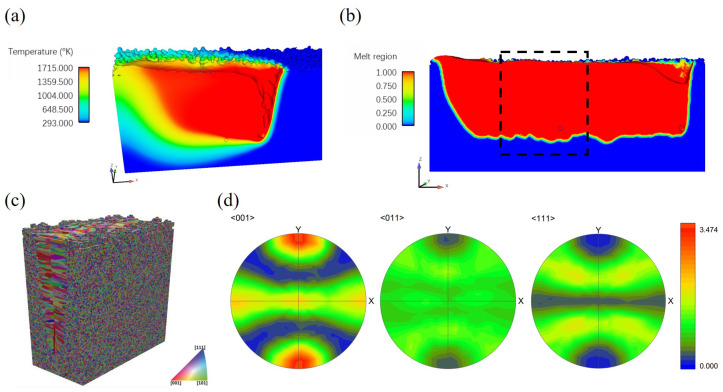
Keyhole-mode melting simulation results: (**a**) deep and narrow melt pool, (**b**) fusion zone with pore defects, where the black dashed line outlines the region for CA simulation, (**c**) 3D view of the microstructure simulation result, (**d**) pole figures of solidification grain structure within the fusion zone.

Under the high-power, low-scan speed laser melting condition, a topological deep-narrow vapor depression is found in the melt pool under the laser spot, as shown in Figure 23a. In addition, the depth–width ratio of the melt pool is 3.5, which is much larger than that of the melt pool with the conduction mode melting, as shown in Section 3.2. This deep–narrow depression is the so-called keyhole. Within the keyhole, the laser beam experiences multiple reflections, improving the laser energy absorption efficiency and further enhancing the metal evaporation to form a deep and narrow hole. As shown in Figure 23a, the keyhole wall fluctuates and is prone to collapsing at the bottom tip due to the complex interplay between thermo-capillary force, Marangoni convection, and recoil pressure [49,50,51]. The instability of the keyhole wall leads to bubbles, a few of which are captured by the solidification front and then become the pore defects, as shown in Figure 23b. Consequently, in keyhole-mode melting, the melt pool with the high depth–width ratio and the presence of pore defects could yield grain structure different from that shown in Section 3.2.

#### 3.3.2. Keyhole-Mode Melting Induced Grain Structure

The region size of interest for grain structure prediction is set to 400 μm × 200 μm × 540 μm, through which a melt pool with the presence of keyhole proceeds as outlined in Figure 23b. A 3D view of the predicted grain structure is plotted in Figure 23c. The pole figures of the solidification microstructure within the deep–narrow melt pool are plotted in Figure 23d, which are different from their counterparts shown in Section 3.2.1. The texture becomes stronger than the single-track case in the conduction mode melting process. In particular, the <001> pole figure, as shown in Figure 23d, indicates that the preferred orientation of many grains is best aligned to the *Y* direction attributed to the unique melt pool morphology and the thermal field features.

**Figure 24:**
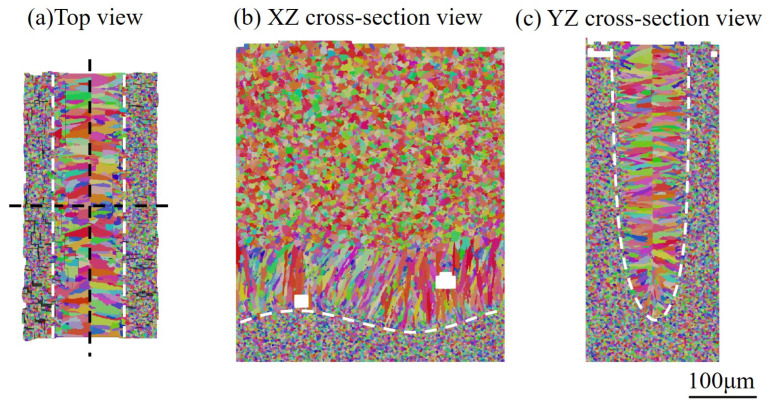
Two-dimensional views of simulation results under the keyhole mode melting: (**a**) top view, (**b**) transverse cross-section map, and (**c**) longitudinal cross-section map, where white dotted lines represent the boundary of the melting zone and black dotted lines represent the location of the cross-section.

In order to detail the influence of keyhole-mode melt pool morphology on microstructure evolution, three 2D views of simulation results are shown in Figure 24. Figure 24a is the top view, which is similar to the result shown in Figure 9a. However, the longitudinal (Figure 24b) and transverse (Figure 24c) cross-section maps display grain structure morphology that is significantly different from that of their counterparts in Figure 9. From the observation of Figure 24b, one can find that the columnar grains from the bottom of the melt pool are blocked by the “equiaxed-like” grains in front of them below one-third of the melt pool depth. As demonstrated in Figure 24c, those “equiaxed-like" grains grow epitaxially from both sides of the deep–narrow melt pool. For the keyhole-mode melting, it is identified that the temperature gradient of both sides of the melt pool projected to the YZ plane is almost aligned with the *Y* direction. Therefore, the grains epitaxially grown from both sides of the melt pool have a growth direction parallel to the *Y* axis. Moreover, the color of these grains is close to red because the grain with orientation best aligned with the temperature gradient can overgrow other grains, as demonstrated by the pole figure shown in Figure 23d. Since the width to depth of the melt pool is large, even greater than three, most of the fusion zone is dominated by the columnar grains from both sides of the melt pool rather than the ones from the melt pool bottom. In addition, the two pores were found in the longitudinal cross-section for the given process parameters, as shown in Figure 24b, which is further addressed in the following subsection.

#### 3.3.3. Effect of Pore Defect on Grain Structure

**Figure 25:**
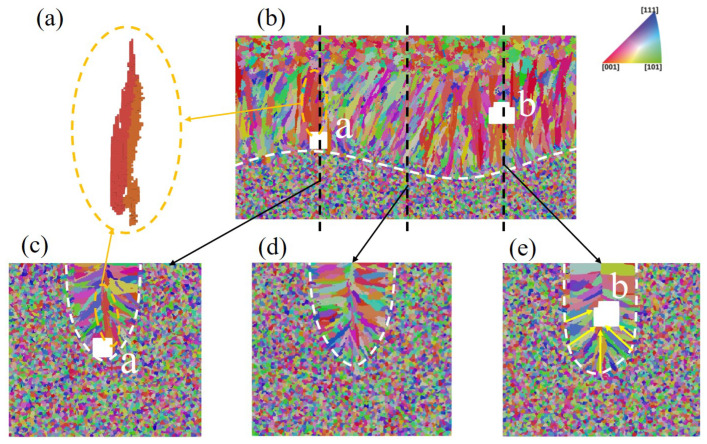
Microstructure near the pores: (**a**) coarse grains above pores *a*, (**b**) longitudinal cross-section map of the grain structure, where the black dotted lines indicate the location of the transverse cross-section maps for (**c**) with the presence of Pore *a* (**d**) without the presence of Pore and (**e**) with the presence of Pore *b*, where white dotted lines represent the boundary of the melting zone and yellow arrow marks several grains blocked by Pore *b*.

A close-up of the grain structure around the two pores, as shown in Figure 24b, is plotted in Figure 25, including the central longitudinal and transverse cross-section views. In Figure 25, the two pores are marked as *a* and *b*. The black dashed lines indicate the location of the transverse cross sections, two grains right above Pore *a* are isolated, and a transverse cross-section without pores is plotted for comparison.

**Figure 26:**
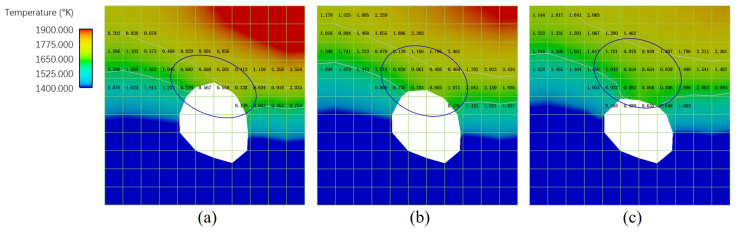
The cooling rate (with the order of $\left(10^{6}\;K/s\right)$) near the mushy zone around pore *a*: (**a**) results at *t* = 9.3 × 10${}^{-4}$ s, (**b**) results at *t* = 9.5 × 10${}^{-4}$ s (**c**) results at *t* = 9.7 × 10${}^{-4}$ s, where the two white curves represent the solidus temperature and liquidus temperature isotherms. The cooling rate of the cells above the pore outlined by the blue curves is smaller than that of the neighboring cells in the mushy zone.

There are two main effects of pores on microstructure evolution. The first is that the pores may block the grain growth from its sides and bottom. From the comparison of Figure 25c,d (without pores), and Figure 25e, it is identified that Pore *a* located very close to the fusion line leads to the absence of epitaxial grain growth from the beneath fusion line; Pore *b*, located far from the melt pool bottom, blocks the growth of grains marked with the arrows in yellow. Consequently, the presence of pores has a significant influence on the local grain structure. The other effect is that the existence of pores can cause changes in the local thermal field, particularly during the cooling stage, and then alter the local grain morphology. Taking Pore *a* as an example, a few snapshots of the thermal field around it with cooling rate marks are plotted in Figure 26. As outlined by the solid blue circle, one can find that the cooling rates of the cells above the Pore are significantly lower than their counterparts in the mushy zone without pores below them. The reduction in cooling rate is attributed to the fact that Pore *a* serves as thermal insulation. With such special thermal field features, coarser grains can be expected above Pore *a*, which is demonstrated in Figure 25a. In addition, Pore *a* blocks the epitaxial growth of grains below it, contributing to the coarser grain growth above Pore *a*.

## 4. Conclusions

This paper conducts a high-fidelity numerical study of the microstructure evolution in selective laser melting 316L stainless steel. The powder scale thermo-fluid flow model using finite volume method and volume of fluid method is adopted to obtain the temperature field, and a 3D cellular automaton model is used to predict the final grain structure, which can link the process details on powder scale with the final microstructure on the micron scale. The calculation accuracy and effectiveness of the numerical model are validated against experimental results. Two sets of numerical examples with different process parameters are simulated to provide insights on grain structure evolution during the conduction mode and keyhole mode melting processes. The main conclusions are as follows.

1. For the conduction mode melting process, it is identified that the grain size increases with the increase in laser power and the decrease in scan speed. For the given ranges, the laser power and scan speed have little effect on the grain structure morphology of the single track. For the given laser power and scan speed, the grain size increases, and the texture gradually becomes strong with the increase in the layers with the unidirectional scan pattern, which can be attributed to the epitaxial grain growth. However, rotating the scan pattern between layers can change the microstructure morphology and weaken the texture.
2. For the keyhole-mode melting process, the obtained grain structure of the single track is significantly different from its counterpart in the conduction mode melting process. Due to the deep–narrow melt pool and the thermal fields within the mushy zone, the fusion zone is dominated by the columnar grains with a growth direction perpendicular to the build direction. These columnar grains epitaxially grow from the sides of the melt pool and block the columnar grains from the bottom of the melt pool. Consequently, the texture of the single track with the keyhole mode melting condition is stronger, and the preferred grain orientations are best aligned to the *Y* direction (perpendicular to the scan and build direction).
3. It is identified that the keyhole-induced pores have two effects on the microstructure evolution. One is to block the grain growth beneath it. The other is to play a role of heat insulation, which reduces the cooling rate above it and thus increases the possibility of forming coarse grains.

## Figures and Tables

**Table 1:** Physical properties of 316L stainless steel [6,42,43,44].

Material Properties	Value
Density, ρ	7650 kg/m${}^{3}$
Solidus temperature, $T_{s}$	1598 K
Liquidus temperature, $T_{r}$	1715 K
Evaporation temperature, $T_{v}$	3090 K
Latent heat of fusion, $L_{m}$	2.7 × 10${}^{5}$ J/kg
Latent heat of vaporization, $L_{v}$	7.45 × 10${}^{6}$ J/kg
Specific heat, $c_{h}$	770.2 J/(kg·K)
Viscosity, μ	0.00345 Pa·s
Surface tension coefficient, σ	1.6 N/m
Temperature coefficient of surface tension, $\frac{d\sigma }{dT}$	−0.00026 N/(m·K)
Molecular mass, *m*	9.3 × 10${}^{-26}$ kg
Boltzmann constant, $k_{B}$	1.3806505 × 10${}^{-23}$ J/K
Convective heat transfer coefficient, $h_{c}$	5.7 W/(m${}^{2}$ K)
Stefan–Boltzmann constant, $\sigma_{s}$	5.67 × 10${}^{-8}$ W/(m${}^{2}$ K${}^{4}$)
Emissivity, ε	0.26
Laser absorption coefficient, $\eta_{0}$	0.4

**Table 2:** Parameters for thermo-fluid flow model.

Parameter	Value
Atmospheric pressure, $p_{0}$	1.013 × 10${}^{5}$ Pa
Ambient temperature, $T_{ref}$	293 K
Laser beam radius, *r*	35 μm [6]
Cell size	4 μm

**Table 3:** Nucleation parameters for the CA model [45].

Parameter	Value
Site density, $\rho_{v}$	1 × 10${}^{6}$ mm${}^{-3}$
Mean undercooling, $T_{N}$	2 K
Standard deviation of undercooling $\Delta T_{\sigma }$	0.5 K
Cell size	1 μm
Growth kinetics parameter, $a_{2}$	2.49 × 10${}^{-7}$ m/(s·K${}^{2}$)
Growth kinetics parameter, $a_{3}$	6.2 × 10${}^{-8}$ m/(s·K${}^{3}$)

**Table 4:** Particle size distribution of 316L stainless steel powder [6].

Diameter (μm)	15	18	21	24	27	30	33
Proportion (%)	15	15	20	18	15	11	6

**Table 5:** Process parameters of the single-track cases (Note).

Case	Laser Power (W)	Scan Speed (m/s)	Layer Thickness (μm)
P140V63	140	0.63	50
P160V63	160	0.63	50
P180V63	180	0.63	50
P200V63	200	0.63	50
P180V53	180	0.53	50
P180V73	180	0.73	50
P180V83	180	0.83	50

**Table 6:** Comparison of melt pool width and depth between experiment data and simulation results for Case P180V63.

	Width	Depth
Experiment data	90 μm	145 μm
Simulation result	87 μm	148 μm
Relative error	3.3%	2.1%

## Data Availability

The main data supporting the findings are available within the article. Additional data are available from the corresponding author upon reasonable request.

## Appendix A. Calculations of *G R M* and *C*

We extracted *G* directly from the thermo-fluid flow simulation result in the central longitudinal cross-section of the mushy zone and calculated *R* as follows.

(A1) R=v·cosθ

where *v* is the scan speed and cosθ reads:

(A2) $$\cos\theta =\frac{\frac{\partial T}{\partial x}}{\sqrt{{\left(\frac{\partial T}{\partial x}\right)}^{2}+{\left(\frac{\partial T}{\partial z}\right)}^{2}}}$$

with θ being the angle between the scan direction and the temperature gradient direction. Based on the values of *G* and *R*, the morphology factor is calculated as M=G/R describing the morphology of the solidification structure, and the cooling rate reads C=G·R, dictating the size of the solidification structure.
